# Trends in healthcare-associated infections and antimicrobial-resistant organisms among adults in Canadian acute care hospitals: findings from four point prevalence surveys, 2002 to 2024

**DOI:** 10.1017/ice.2025.10259

**Published:** 2025-10

**Authors:** Robyn Mitchell, Diane Lee, Jessica Bartoszko, Cassandra Lybeck, Marie-Ève Benoit, Jeannette Comeau, Jennifer Ellison, Charles Frenette, Jennifer Happe, Nicole Haslam, Bonita Lee, Dominik Mertz, Stephanie W. Smith, Daniel Thirion, Alice Wong, Michelle Science, Susy Hota

**Affiliations:** 1 Canadian Nosocomial Infection Surveillance Program, Public Health Agency of Canada, Ottawa, Ontario, Canada; 2 Infection Control Department, CHU Sainte-Justine, Montréal, Québec, Canada; 3 Department of Pediatrics, Dalhousie University, Halifax, Nova Scotia, Canada; 4 Infection Prevention and Control, Alberta Health Services, Calgary, Alberta, Canada; 5 Department of Medicine, McGill University Health Centre, Montréal, Québec, Canada; 6 Infection Prevention and Control, Health Sciences North, Sudbury, Ontario, Canada; 7 Department of Pediatrics, Stollery Children’s Hospital, Edmonton, Alberta, Canada; 8 Infection Prevention and Control, Hamilton Health Sciences, Hamilton, Ontario, Canada; 9 Faculty of Medicine, University of Alberta, Edmonton, Alberta, Canada; 10 Faculty of Pharmacy, McGill University Health Centre, Montréal, Québec, Canada; 11 Department of Medicine, Division of Infectious Diseases, Royal University Hospital, Saskatoon, Saskatchewan, Canada; 12 Infection Prevention and Control, Hospital for Sick Children, Toronto, Ontario, Canada; 13 Department of Medicine, University Health Network, Toronto, Ontario, Canada

## Abstract

**Objective:**

To describe trends in the prevalence of healthcare-associated infections (HAIs) and antibiotic-resistant organisms (AROs) in Canadian acute-care hospitals.

**Design:**

Repeated point prevalence surveys.

**Setting:**

Canadian Nosocomial Infection Surveillance Program (CNISP) hospitals.

**Methods:**

Trained infection control professionals reviewed medical records of eligible adult patients and applied standardized definitions to collect demographic data and information on HAIs, AROs, and additional precautions from 39 to 62 hospitals in 2002, 2009, 2017, and 2024.

**Results:**

The prevalence of adult patients with at least one HAI increased from 10.4% (95% CI: 9.6%–11.2%) in 2002 to 12.4% (95% CI: 11.7%–13.2%) in 2009, declined to 8.4% (95% CI: 7.8%–9.0%) in 2017, and stabilized in 2024 (8.1%, 95% CI: 7.6%–8.6%) despite 3.1% of HAIs being due to SARS-CoV-2. Between 2017 and 2024, there were increases in bloodstream infections (1.0% to 1.5%, *p* = 0.002), viral respiratory infections (VRI) (0.3% to 0.6%, *p* < 0.001), and in the prevalence of patients on additional precautions for carbapenemase-producing organisms (0.1% to 1.7%, *p* < 0.001) and VRIs (2.1% to 3.6%, *p* < 0.001). In 2024, AROs were responsible for 6.6% of infections. One-third of HAIs were device-associated, and the prevalence of central line-associated bloodstream infections (CLABSIs) doubled from 0.4% in 2017 to 0.7% in 2024, *p* = 0.02.

**Conclusions:**

A point prevalence survey performed in Canada in 2024 following the COVID-19 pandemic identified a stable prevalence of HAIs and AROs despite the inclusion of SARS-CoV-2. Concerning trends were observed including the increased prevalence of certain HAIs such as CLABSIs and VRIs highlighting the need for ongoing efforts in hospital infection prevention.

## Introduction

Healthcare-associated infections (HAIs) and antimicrobial resistance pose an increasing threat to global health.^1,2^ Point prevalence surveys are a key surveillance tool that can inform hospital infection prevention and control (IPAC) programs on the overall burden and trends of HAIs and antimicrobial-resistant organisms (AROs) and influence associated IPAC mitigation practices. They are a useful low-cost alternative to active surveillance and provide valuable benchmarking data across healthcare settings and jurisdictions.^3^

Several countries^4–9^ perform repeated point prevalence surveys to compliment prospective data and describe evolving IPAC practices. While direct comparison of results across countries is challenging due to the variability of survey methodology and differences in healthcare systems,^10^ common findings have been identified. These include a higher prevalence of HAIs among patients in intensive care, high utilization of invasive devices and device-associated infections as well as the emergence of resistant pathogens, highlighting the need for vigilant and improved infection control strategies.

The Canadian Nosocomial Infection Surveillance Program (CNISP) previously performed three point prevalence surveys of HAIs, AROs, and antimicrobial use (AMU). The prevalence of patients with at least one HAI increased from 9.9% (2002) to 11.3% (2009) then declined to 7.9% (2017).^11^ Results showed that one in 12 patients (and one in eight patients admitted to intensive care) had a HAI and that AROs were responsible for 9% of these infections.^12^

In 2024, CNISP conducted a fourth national point prevalence survey. We provide an updated estimate of the prevalence of HAIs, AROs, and isolation burden among adult inpatients in Canadian acute care hospitals following the COVID-19 pandemic and identify trends across the four surveys.

## Methods

### Study design

Point prevalence surveys were conducted in CNISP acute-care hospitals in 2002, 2009, 2017, and 2024. Survey methodology has been published elsewhere.^11,13^

### Study population

CNISP is a collaboration between the Public Health Agency of Canada, the National Microbiology Laboratory, the Association of Medical Microbiology and Infectious Disease Canada, and sentinel hospitals across Canada that conduct standardized surveillance of HAIs and AROs.^14^

Patients of any age who were admitted to a participating CNISP hospital for 48 hours or longer were eligible for inclusion, including admitted patients in the emergency department. Patients who had been admitted for less than 48 hours but were admitted within the last month to the survey hospital were also included. We excluded patients admitted to long-term care, maternity, mental health, day surgery, or rehabilitation units.

### Definitions

National Healthcare Safety Network (NHSN) HAI definitions were used for pneumonia, urinary tract infection (UTI), surgical site infection (SSI), and viral gastroenteritis.^15^ Bloodstream infection (BSI), *Clostridioides difficile* infection (CDI), and viral respiratory infection (VRI) were defined using CNISP definitions.^16,17^

We considered a HAI to be present if the patient was symptomatic of, or was receiving antimicrobial therapy for, an infection on the day of the survey and the onset of symptoms was on day 3 (≥48 hours) or later of hospital admission. For healthcare-associated (HA) VRI and CDI, symptom onset was on day 4 (≥72 hours) or later of hospital admission. If a SSI was present on the day of the survey or the patient was being treated for a SSI on the day of the survey, and SSI definition criteria were met, this was captured as a HAI.

### Training, data collection, and validation

Survey methodology and standardized forms were developed and piloted by an expert working group in 2002 and updated accordingly for each survey year. The surveys were conducted in February or March of each survey year to limit the influence of seasonal variation in HAIs and to permit comparison between surveys. Training on the survey methodology was provided to IPAC leads within each CNISP hospital. IPAC leads trained their frontline IPAC staff on definitions, data collection, and data entry procedures. IPAC staff reviewed the medical records of eligible patients (patients with or without a HAI) and data were submitted electronically through a secure online web-based platform or by a standardized Excel template.

We collected de-identified patient-level data, including demographics, additional precautions, and the presence of HAI, ARO, and AMU. AMU data will be reported separately. In 2024, a subset of hospitals (*n* = 32) collected data on the utilization of invasive devices. Data variables collected in each survey are outlined in Supplementary Table 1. CNISP collects hospital-level data (e.g., number of beds and specialized services provided) annually using a standardized hospital profile form. We extracted hospital profile data for CNISP hospitals that participated in the four surveys and included these data in the analyses.

CNISP epidemiologists evaluated the data for quality and completeness and followed up with hospitals as needed. To assess the level of agreement between primary and validation data collectors, a validation survey was piloted among 472 patients in 41 hospitals. Validation methods are further described^18^ and full results will be reported separately.

### Statistical analysis

Analyses were conducted using R statistical software version 4.3.2.^19^ We compared the characteristics of participating hospitals and patients who were surveyed, the prevalence of HAIs and AROs causing infection using *χ* ^2^ tests, Fisher’s exact tests for categorical variables, or Kruskal–Wallis tests for continuous variables. The Mann-Kendall test was used to test trends. Agreement between the data collections was analyzed using kappa (κ) statistics (0.81–1.00 very good, 0.61–0.80 good, 0.41–0.60 moderate, 0.21–0.40 fair/marginal, and <0.2 poor). We considered a 2-sided *P* value ≤ 0.05 to be statistically significant.

Analyses were restricted to adult patients (≥18 years). We calculated the prevalence of HAIs as the percentage of patients with at least one HAI on the survey day over the total number of patients surveyed and 95% confidence intervals (CIs) were calculated. We previously reported that characteristics of the participating hospitals were similar over the 2002, 2009, and 2017 surveys.^11^ To account for changes in the hospital characteristics between the 2017 and 2024 surveys, we conducted a sensitivity analysis restricted to the 39 hospitals that participated in both of these surveys.

### Ethical considerations

These surveys were either considered exempt as quality assurance projects or approved by the research ethics boards at participating hospitals.

## Results

### Hospital and patient characteristics

In 2024, 62 of 109 CNISP hospitals participated in the point prevalence survey (response rate 56.9%), which included 10,473 eligible adult patients.

The characteristics of the participating hospitals were compared across the four surveys (Supplementary Table 2). Compared to previous surveys, in 2024 we observed a decrease in the median bed size, the proportion of teaching hospitals, and the proportion of hospitals which provide the following specialized services: intensive care, burn, and solid organ transplant. This change in hospital characteristics is primarily reflective of the addition of small, community hospitals in recent years to improve the representativeness of the CNISP network.

The characteristics of adult patients surveyed were compared across all four surveys (Supplementary Table 3). A difference in patient age, sex, and location in hospital were reported, though the magnitude of these differences was small from a clinical perspective.

### Prevalence of HAIs

In 2024, among 10,473 patients surveyed, 844 (8.1%) were reported to have at least one HAI, with a total of 913 HAIs reported. Of those, 779 (7.4%) patients had one HAI, 61 (0.6%) had two HAIs, and 4 (<0.1%) had three or more HAIs.

The prevalence of patients with at least one HAI increased from 10.4% (95% CI: 9.6%–11.2%) in 2002 to 12.4% (95% CI: 11.7%–13.2%) in 2009 followed by a decline to 8.4% (95% CI: 7.8%–9.0%) in 2017. HAI prevalence remained stable in 2024 at 8.1% (95% CI: 7.6%–8.6%). After excluding COVID-19 infections in 2024, the HAI prevalence remained similar (7.8%, 95% CI: 7.3%–8.3%). In an analysis restricted to the 39 hospitals which participated in both the 2017 and 2024 surveys, the prevalence of infection remained stable between the 2017 (8.3%, 95% CI: 7.6%–8.9%) and 2024 (8.0%, 95% CI: 7.4%–8.6%) surveys. The validation results found a 93.2% agreement (*κ* = 0.62) between the primary and validation results for the presence of a HAI. The level of agreement ranged from *κ* = 0.58 for HA-pneumonia to *κ* = 0.72 for HA-CDI.

**Figure 1.**
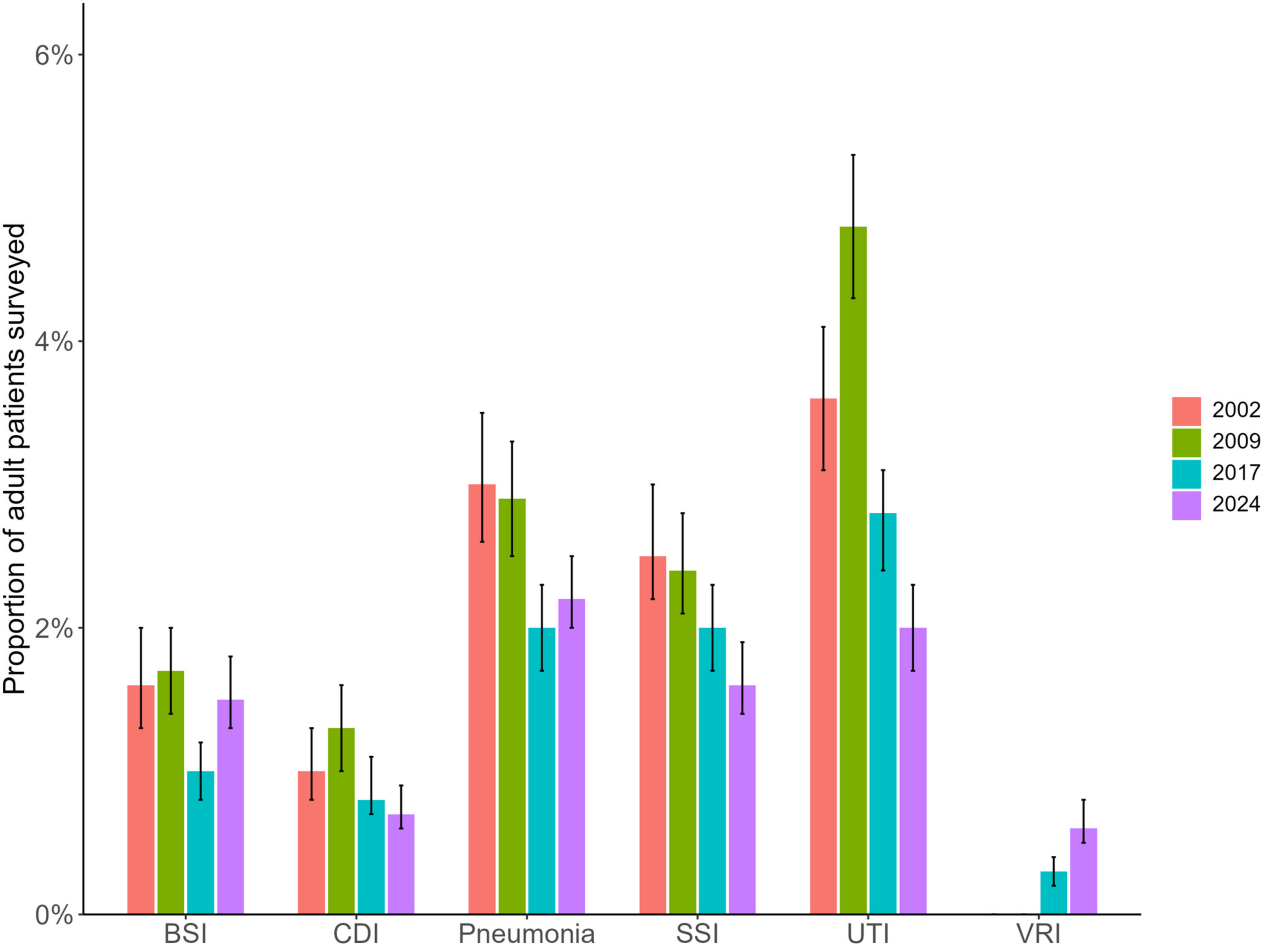
Prevalence of healthcare-associated infection types among adult patients surveyed in 2002, 2009, 2017, and 2024. *Note:* BSI = bloodstream infection, CDI = *Clostridioides difficile* infection, SSI = surgical site infection, UTI = urinary tract infection, VRI = viral respiratory infection.

From 2002 to 2024, the prevalence of patients with a SSI or UTI declined. The prevalence of patients with pneumonia or CDI decreased from 2009 to 2017 and remained stable in 2024. The prevalence of patients with a primary BSI significantly declined from 1.7% (95% CI: 1.4%–2.0%) in 2009 to 1.0% (95% CI: 0.8%–1.2%) in 2017 then significantly increased to 1.5% (95% CI: 1.3%–1.8%) in 2024 (*p* = 0.002). The prevalence of patients with a VRI while low, significantly increased from 0.3% (95% CI: 0.2%–0.4%) in 2017 to 0.6% (95% CI: 0.5%–0.8%) in 2024 (*p* < 0.001) (Figure 1, Supplementary Table 4).

For all four surveys combined, the prevalence of HAIs was significantly higher in patients admitted to ICU, where 24.7% (95% CI: 22.9%–26.7%) had at least one HAI compared with 8.6% (95% CI: 8.2%–8.9%) in all other units combined (*p* < 0.001). We observed a decline in the prevalence of HAIs in patients in the ICU from 35.5% (95% CI: 30.1%–41.3%) in 2002 to 29.6% (95% CI: 25.6%–34.0%) in 2009, 20.2% (95% CI: 17.1%–23.8%) in 2017, then stabilization in 2024 (20.6%, 95% CI: 17.7%–23.8%). The prevalence of ICU patients with a UTI, SSI, and pneumonia significantly declined over time. The prevalence of ICU patients with CDI remained unchanged. The prevalence of ICU patients with a BSI declined from 2002 to 2017 followed by a significant increase in 2024 (Supplementary Table 4).

### Prevalence of invasive devices and device-associated infections

Among the subset of 32 hospitals which collected data on the use of invasive devices in 2024, 38.0% (2,526/6,644) of patients surveyed had at least one invasive device on the survey day. Indwelling urinary catheter (18.1%, *n* = 1204) and central venous catheter were the most common (17.7%, *n* = 1176) followed by inserted tubes and drains (15.0%, *n* = 998) and endotracheal intubation (4.4%, *n* = 295). All of the above invasive devices were more common among patients in ICU compared to patients in coronary care, medical, and surgical units (Supplementary Table 5).

Among patients surveyed, the prevalence of catheter-associated infection, ventilator-associated pneumonia, and SSI associated with a prosthetic implant remained similar between the 2017 and 2024 surveys. However, the prevalence of central line-associated BSIs while low, nearly doubled between the 2017 (0.4%) and 2024 (0.7%, *p* = 0.02) surveys (Supplementary Table 4).

### Prevalence of additional precautions

The prevalence of patients on additional precautions significantly increased from 2017 (15.4%, 95% CI: 14.7%–16.2%) to 2024 (21.7%, 95% CI: 20.9%–22.5%, *p* < 0.001). This increase was mainly driven by an increase in contact precautions from 14.0% (95% CI: 13.3%–14.7%) in 2017 to 20.2% (95% CI: 19.5%–21.0%) in 2024 (*p* < 0.001) and to a lesser extent an increase in the proportion of patients on droplet/contact precautions (2.7%, 95% CI: 2.4%–3.1% to 4.3%, 95% CI: 3.9%–4.7%, *p* < 0.001).

**Fig. 2.**
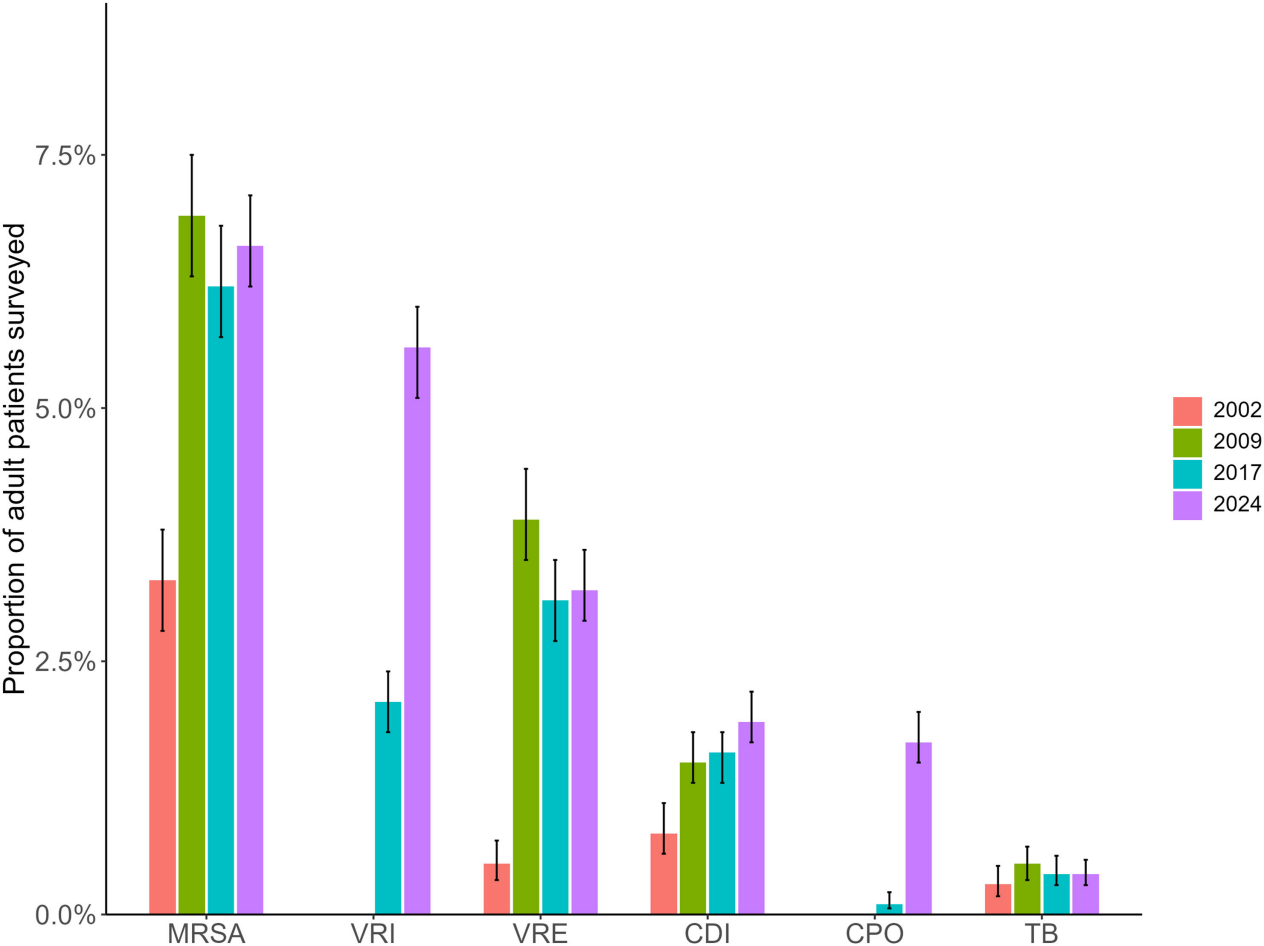
Prevalence of adult patients on additional precautions, 2002, 2009, 2017, and 2024. *Note:* MRSA = methicillin-resistant *S. aureus*, VRI = viral respiratory infection, VRE = vancomycin-resistant Enterococci, CDI = *Clostridioides* difficile infection, CPO = carbapenamase-producing organisms, TB = Tuberculosis.

Among patients surveyed in 2024, the most common reason for additional precautions was methicillin-resistant *Staphylococcus aureus* (MRSA) (6.6%, 95% CI: 6.2%–7.1%), followed by VRI (5.6%, 95% CI: 5.1%–6.0%) of which 2.4% (95% CI: 2.1%–2.7%) were COVID-19, vancomycin-resistant Enterococci (VRE) (3.2%, 95% CI: 2.9%–3.6%), CDI (1.9%, 95% CI: 1.7%–2.2%), and carbapenemase-producing organisms (CPO) (1.7%, 95% CI: 1.5%–2.0%). In addition, based on risk assessment upon admission three patients were isolated for *Candida auris* in 2024. Between 2017 and 2024, we observed a significant increase in the prevalence of patients isolated for CPO (0.1% to 1.7%, *p* < 0.001) and VRI (2.1% to 5.6%, *p* < 0.001) (Figure 2).

### Prevalence of antimicrobial-resistant organisms

Table 1 summarizes select AROs that cause HAIs. Overall, AROs remain an uncommon cause of HAIs across all survey years. The most common resistant organism was MRSA which was present in 5.8% of pneumonia infections, 5.6% of BSIs, 4.0% of SSIs, and 1.2% of UTIs. Carbapenemase-producing *Enterobacterales* (CPE) were identified in only five infections in the 2017 and 2024 surveys.

**Table 1.** Selected antimicrobial-resistant organisms causing healthcare-associated infections in 2002, 2009, 2017, and 2024

Type of infection	No. (%) of infections					*P* value*
	2002	2009	2017	2024	Total (2002-2024)	
**Urinary tract infections, no (%)**	no. (%)	no. (%)	no. (%)	no. (%)	no. (%)	
MRSA	2 (1.0)	4 (1.1)	4 (1.8)	3 (1.4)	13 (1.3)	0.89
VRE	2 (1.0)	4 (1.1)	3 (1.4)	4 (1.8)	13 (1.3)	0.86
CPE	n/a	n/a	2 (0.9)	1 (0.5)	3 (0.3)	1.0
ESBL	0 (0.0)	16 (4.5)	12 (5.4)	8 (3.7)	36 (3.6)	**0.002**
Total UTI organisms	210	355	221	217	1003	
**Pneumonia, no (%)**						
MRSA	10 (7.0)	11 (7.4)	8 (7.3)	8 (6.3)	37 (7.0)	0.99
ESBL	0 (0.0)	1 (0.7)	2 (1.8)	3 (2.4)	6 (1.1)	0.20
Total pneumonia organisms	143	149	110	127	529	
**Surgical site infections, no (%)**						
MRSA	11 (7.6)	7 (4.1)	9 (5.4)	4 (2.1)	31 (4.6)	0.12
VRE	0 (0.0)	1 (0.6)	2 (1.2)	5 (2.7)	8 (1.2)	0.15
CPE	n/a	n/a	1 (0.6)	0 (0.0)	1 (0.1)	0.47
ESBL	2 (1.4)	1 (0.6)	4 (2.4)	5 (2.7)	12 (1.8)	0.45
Total SSI organisms	145	171	168	187	671	
**Bloodstream infections, no (%)**						
MRSA	4 (5.6)	5 (4.5)	9 (10.5)	8 (5.3)	26 (6.2)	0.35
VRE	0 (0.0)	1 (0.9)	2 (2.3)	5 (3.3)	8 (1.9)	0.36
CPE	n/a	n/a	0 (0.0)	1 (0.7)	1 (0.2)	1.0
ESBL	1 (1.4)	4 (3.6)	0 (0.0)	5 (3.3)	10 (2.4)	0.31
Total BSI organisms	72	112	86	152	422	
**All infections, no (%)**						
MRSA	27 (4.7)	27 (3.4)	30 (5.1)	23 (3.1)	107 (4.1)	0.18
VRE	2 (0.4)	6 (0.8)	7 (1.2)	14 (1.9)	29 (1.1)	0.05
CPE	n/a	n/a	3 (0.5)	2 (0.3)	5 (0.2)	0.67
ESBL	3 (0.5)	22 (2.8)	18 (3.0)	21 (2.8)	64 (2.4)	**0.003**
**Total organisms**	570	792	594	747	2703	

MRSA = methicillin-resistant *S. aureus*, VRE = vancomycin-resistant Enterococci, CPE = carbapenamase-producing *Enterobacterales*, ESBL = extended-spectrum β-lactamase–producers, SSI = surgical site infections, BSI = bloodstream infections, UTI = urinary tract infection, n/a = not available (data not collected). * The Mann-Kendall test was used to test trends.

The prevalence of infections with extended-spectrum β-lactamase–producers significantly increased between 2002 (0.4%) and 2024 (2.6%) (*p* = 0.001) and were most common in patients with UTIs and BSIs. While VRE infrequently caused a HAI, the prevalence of infections associated with VRE significantly increased from 0.3% in 2002 to 1.7% in 2024 (*p* = 0.02).

### Microbiology

In the 2024 survey, a total of 747 organisms were reported for 81.8% (913) of HAIs, mostly gram-negative organisms (48.3%), followed by gram-positive organisms (37.2%), viruses (8.7%), and fungi (5.9%). The most frequently isolated organisms for HAIs were *E. coli* (13.8%) and *Enterococcus spp.* (12.3%) both of which were most commonly identified in UTIs. *S. aureus* was a frequent cause of BSIs, SSIs, and pneumonia. *Klebsiella spp*. was most commonly identified in UTIs and *Pseudomonas spp.* was a common cause of pneumonia and SSIs (Table 2). An important emerging organism, SARS-CoV-2, accounted for 3.1% of all HAIs and nearly half of all VRIs. *Candida auris* was not identified in the 2024 survey.

**Table 2.** Microorganisms isolated in HAIs by infection type, 2024

Microorganism	Number	All HAIs, %	Pneumonia No. (%)		Urinary tract infection No. (%)		Surgical site infection No. (%)		Bloodstream infection No. (%)		Viral respiratory infection No. (%)	
*E. coli*	126	13.8	4	1.7	81	38.8	19	11.2	22	13.8	0	0
*Enterococcus spp.*	112	12.3	0	0	59	28.2	33	19.4	20	12.6	0	0
*Staphylococcus aureus*	95	10.4	33	14.2	2	1.0	26	15.3	34	21.4	0	0
*Klebsiella spp.*	64	7.0	15	6.5	27	12.9	10	5.9	12	7.6	0	0
*Pseudomonas spp.*	55	6.0	23	9.9	11	5.3	16	9.4	5	3.1	0	0
*Candida spp.*	41	4.5	10	4.3	8	3.8	13	7.7	10	6.3	0	0
*Enterobacter spp.*	35	3.8	8	3.5	10	4.8	10	5.9	7	4.4	0	0
SARS-CoV-2	28	3.1	0	0	0	0	0	0	0	0	28	43.1
Other *Staphylococcus spp.*	30	3.3	1	0.4	0	0	11	6.5	18	11.3	0	0
*Proteus spp.*	18	2.0	2	0.9	10	4.8	5	2.9	1	0.6	0	0
*Streptococcus spp.*	26	2.9	0	0	0	0	13	7.7	13	8.2	0	0
Other	117	12.9	31	13.4	9	4.3	31	18.2	10	6.3	32	49.2

Other microorganisms include: Coagulase-negative staphylococci, *Stenotrophomonas maltophilia*, *H. influenzae*, norovirus, rhinovirus, influenza, respiratory syncytial virus, adenovirus*, Serratia spp*., *Citrobacter spp., Acinetobacter spp*. etc.

With the exception of SARS-CoV-2, the distribution of the most frequently identified microorganisms was similar to the findings of the previous surveys (Supplementary Table 6).

In the analysis restricted to the 39 hospitals that participated in both the 2017 and 2024 surveys, we found that the prevalence of device-associated infections, additional precautions, AROs, and organisms were similar compared to the primary analysis which included all hospitals surveyed.

## Discussion

We report the prevalence of HAIs, AROs, and isolation burden among adult patients in a network of Canadian acute-care hospitals based on findings from four repeated point prevalence surveys in 2002, 2009, 2017, and 2024. The prevalence of HAIs among patients remained stable between 2017 (8.4%) and 2024 (8.1%), despite 3.1% of HAIs being due to SARS-CoV-2 in 2024. Among infection types surveyed, the prevalence of UTIs and SSIs declined over time. However prior success in a reduction of the prevalence of pneumonia and CDI were not sustained and an increase in the prevalence of BSIs and VRIs were observed.

While we did not measure the impact of the COVID-19 pandemic on the prevalence of HAIs and AROs in our 2024 survey, a time-series analysis conducted among CNISP hospitals identified that the COVID-19 pandemic was associated with surveillance trends similar to those reported in our surveys.^21^ Furthermore, findings from our 2024 survey were similar to results from other surveys performed either during or post the COVID-19 pandemic. In 2021, New Zealand reported a HAI prevalence of 6.6% among adult patients.^9^ In 2022, the prevalence of HAIs among all patients in Greece^21^ and Italy^22^ was 10.6% and 7.4%, respectively. The ECDC reported a HAI prevalence of 8.0% in their 2022–2023 survey among 31 countries^23^ and England reported a prevalence of infection of 7.6% among patients of all ages in 2023.^24^ The variation in international prevalence may reflect a real difference in the prevalence of HAIs or differences in healthcare systems (e.g., patient severity and comorbidities and hospital characteristics), methodology (e.g., hospital types, inclusion criteria, and case definitions), or the timing of surveys with respect to the COVID-19 pandemic.

Overall, AROs remain an uncommon cause of HAIs across Canada. We collected data on CPE in the 2017 and 2024 surveys and identified only five infections. However, the prevalence of patients on additional precautions for CPO significantly increased between 2017 (0.1%) and 2024 (1.7%). Furthermore, national surveillance data indicates that while the incidence of CPE remains low a recent exponential increase has been identified.^25^ These findings all serve as a warning of the impact of increasing rates of CPE in Canadian hospitals. Nearly half of HAIs reported in 2024 were associated with gram-negative organisms from which the greatest threat of antimicrobial resistance currently exists.

There were no cases of HA *C. auris* infection reported among patients surveyed in 2024 despite the global spread of this antimicrobial resistance threat. Three patients were isolated for *C. auris* based on risk factors at admission; with the variability of screening practices across Canada it is possible that some colonized patients were not identified.^26^ We identified a significant increase in the prevalence of patients on additional precautions for VRIs (1.7% in 2017 to 5.6% in 2024). This reflects the high seasonal burden of respiratory viruses on Canadian hospitals.

There was no significant reduction in the prevalence of infection among patients in the ICU from 2017 (20.2%) to 2024 (20.6%); however, the burden remains high as one in five ICU patients developed a HAI in 2024. These findings are consistent with other countries which have recently reported a high prevalence of infection among patients in the ICU, ranging from 20.5% to 23%.^9,22,23^ Importantly, we observed a significant increase in the prevalence of BSIs among these high-risk patients which has been identified in other studies during the same time period.^27^ This is of concern as BSIs among ICU patients are associated with substantial morbidity and mortality and antimicrobial resistance further complicates the care of these patients.^28^

In 2024, approximately one-third of all HAIs were device-associated (33%), similar to the 2017 survey (32%) and results from the 2022–2023 European survey (32%).^23^ One-third of all healthcare-associated pneumonia infections and nearly half of all UTIs and BSIs were device-associated. Efforts to implement and improve compliance with evidence-based infection control interventions (e.g., bundles, surveillance, and education) to reduce device-associated HAIs should continue, yet given that two-thirds of HAIs in our survey were not associated with a device or surgical procedure, other intervention strategies are required to reduce their burden. Other strategies may include continued efforts to improve hand hygiene, improving environmental cleaning and disinfection, adopting antimicrobial stewardship principles, and improving IPAC staffing and resources. In addition, support for further research that provides evidence for prevention strategies is needed.^23,29–34^

Limitations of this work include the potential for selection bias as only those hospitals participating in the CNISP network were eligible to participate. However, the network represents approximately 37% of acute care beds in Canada and the addition of small community hospitals has improved the generalizability of these results.^14^ Minor revisions to the surveillance definitions and CDI laboratory testing practices used in previous surveys have been described.^11^ However, as standardized methodology was applied, these revisions had minimal impact on our ability to compare prevalence over time. Our survey findings do not account for the burden of community-associated infections on hospitals; however, these data are captured for some infections through CNISP routine incidence surveillance. Finally, these results were not corrected for changes in patient case mix and hospital characteristics. Therefore, results should be interpreted with caution as hospital participation varied over the surveys. Notably, when we restricted our analysis to only those hospitals which participated in the 2017 and 2024 surveys, we found similar results.

In conclusion, a point prevalence survey performed among a network of Canadian acute-care hospitals in 2024 following the COVID-19 pandemic identified a stable prevalence of HAIs and AROs compared to the 2017 survey, despite the emergence of SARS-CoV-2. These findings provide evidence of national progress in the prevention of HAIs and AROs and underscores the importance of continued investment in hospital infection prevention. Of concern is the increasing trend in BSIs and isolation burden due to CPOs and VRIs which have a high impact on patients and informs future direction for IPAC programs. Preventing and controlling HAIs and AROs will not only improve patient outcomes but it will also preserve the effectiveness of antibiotics and protect our ability to treat infections in the future.

## Supporting information

Mitchell et al. supplementary material Mitchell et al. supplementary material

## Supplementary material

To view supplementary material for this article, please visit https://doi.org/10.1017/ice.2025.10259.
