# Supplementary material for: Trends in healthcare-associated infections and antimicrobial-resistant organisms among adults in Canadian acute care hospitals: findings from four point prevalence surveys, 2002 to 2024
Source: Infect Control Hosp Epidemiol. 2025 Sep 10;46(10):984–91. doi: 10.1017/ice.2025.10259 (PMC12615124; doi:10.1017/ice.2025.10259)
Supplement: Mitchell et al. supplementary material [file S0899823X25102596sup001.docx]

**Supplementary Table 1.** Summary of data collection by survey year (2002, 2009, 2017 and 2024)

|  | **2002** | **2009** | **2017** | **2024** |
| --- | --- | --- | --- | --- |
| **HAIs** |  |  |  |  |
| HA-pneumonia | ✓ | ✓ | ✓ | ✓ |
| HA-UTI | ✓ | ✓ | ✓ | ✓ |
| HA-BSI | ✓ | ✓ | ✓ | ✓ |
| HA-SSI | ✓ | ✓ | ✓ | ✓ |
| HA-CDI | ✓ | ✓ | ✓ | ✓ |
| HA-VRI | ✓ (pediatrics only) | ✓ (pediatrics only) | ✓ | ✓ |
| HA-viral gastroenteritis | ✓ (pediatrics only) | ✓ (pediatrics only) | ✓ | ✓ |
| **Device-associated infections** |  |  |  |  |
| VAP |  |  | ✓ | ✓ |
| CLABSI |  |  | ✓ | ✓ |
| SSI associated with implant |  |  | ✓ | ✓ |
| CAUTI |  |  | ✓ | ✓ |
| **Additional precautions** |  |  |  |  |
| Contact | ✓ | ✓ | ✓ | ✓ |
| Droplet/contact | ✓ | ✓ | ✓ | ✓ |
| Airborne | ✓ | ✓ | ✓ | ✓ |
| Droplet/contact + N95 |  |  |  | ✓ |
| **Reason for additional precautions** |  |  |  |  |
| MRSA | ✓ | ✓ | ✓ | ✓ |
| VRE | ✓ | ✓ | ✓ | ✓ |
| CDI | ✓ | ✓ | ✓ | ✓ |
| Tuberculosis | ✓ | ✓ | ✓ | ✓ |
| ESBL | ✓ | ✓ | ✓ | ✓ |
| CPO |  |  | ✓ | ✓ |
| VRI (not COVID-19) |  |  | ✓ | ✓ |
| COVID-19 |  |  |  | ✓ |
| **AROs** |  |  |  |  |
| MRSA | ✓ | ✓ | ✓ | ✓ |
| VRE | ✓ | ✓ | ✓ | ✓ |
| ESBL | ✓ | ✓ | ✓ | ✓ |
| CPO |  |  | ✓ | ✓ |
| *C. auris* |  |  |  | ✓ |
| **Invasive device utilization** |  |  |  |  |
| Indwelling urinary catheter |  |  |  | ✓ |
| Central vascular catheter |  |  |  | ✓ |
| Inserted tubes and drains |  |  |  | ✓ |
| Invasive endotracheal intubation |  |  |  | ✓ |

BSI = bloodstream infection, CDI = *Clostridioides difficile* infection, SSI = surgical site infection, UTI = urinary tract infection, VRI = viral respiratory infection; CAUTI = catheter-associated urinary tract infection; VAP = Ventilator-associated pneumonia; CLABSI = central line associated bloodstream infection; SSI with implant = surgical site infection associated with a prosthetic implant, CPE = carbapenamase-producing Enterobacterales, ESBL = extended-spectrum β-lactamase–producers, MRSA = methicillin-resistant S. aureus, n/a = not available (data not collected), UTI = urinary tract infection; VRE = vancomycin-resistant Enterococci.

**Supplementary Table 2.** Selected characteristics of participating hospitals for the point prevalence surveys (2002, 2009, 2017 and 2024)

| **No. (%) of hospitals** | | | | | |
| --- | --- | --- | --- | --- | --- |
| **Characteristic** | **2002 N=28** | **2009 N=39** | **2017 N=47** | **2024 N=62** | ***P* value** |
| **Region** |  |  |  |  | 0.93 |
| Western | 10/28 (36%) | 15/39 (38%) | 18/47 (38%) | 19/62 (31%) |  |
| Central | 12/28 (43%) | 16/39 (41%) | 16/47 (34%) | 26/62 (42%) |  |
| Eastern | 6/28 (21%) | 8/39 (21%) | 13/47 (28%) | 17/62 (27%) |  |
| **Hospital bed size** |  |  |  |  | 0.025 |
| Median (IQR) | 441 (260, 600) | 342 (173, 466) | 290 (206, 436) | 225 (114, 428) |  |
| **Hospital type** |  |  |  |  | 0.96 |
| Adult | 13/28 (46%) | 20/39 (51%) | 23/47 (49%) | 34/62 (55%) |  |
| Mixed | 9/28 (32%) | 12/39 (31%) | 17/47 (36%) | 17/62 (27%) |  |
| Pediatric | 6/28 (21%) | 7/39 (18%) | 7/47 (15%) | 11/62 (18%) |  |
| **Teaching hospital** |  |  |  |  |  |
| Yes | 28/28 (100%) | 36/39 (92%) | 41/47 (87%) | 47/62 (76%) | 0.007 |
| **Specialized services** |  |  |  |  |  |
| ICU | 28/28 (100%) | 37/39 (95%) | 44/47 (94%) | 51/62 (82%) | 0.028 |
| Hematology/Oncology | 19/28 (68%) | 26/39 (67%) | 27/47 (57%) | 34/62 (55%) | 0.52 |
| Dialysis | 18/28 (64%) | 28/39 (72%) | 31/47 (66%) | 35/62 (56%) | 0.45 |
| Burn unit | 16/28 (57%) | 14/39 (36%) | 16/47 (34%) | 15/62 (24%) | 0.026 |
| Solid organ transplant | 15/28 (54%) | 16/39 (41%) | 16/47 (34%) | 14/62 (23%) | 0.028 |

IQR = interquartile range; ICU = intensive care unit; Western Canada includes British Columbia, Alberta, Saskatchewan and Manitoba; Central Canada includes Ontario, Quebec and Nunavut; Eastern Canada includes Nova Scotia, New Brunswick, Prince Edward Island and Newfoundland and Labrador

**Supplementary Table 3.** Selected characteristics of adult patients who were surveyed in 2002, 2009, 2017 and 2024

| **Characteristic** | **2002 N=5,773** | **2009 N=7,578** | **2017 N=8,505** | **2024 N=10,473** | ***P* value** |
| --- | --- | --- | --- | --- | --- |
| **Male** | 2948/5773 (51%) | 3,869/7552 (51%) | 4447/8461 (53%) | 5539/10447 (53%) | 0.02 |
| **Age, year** |  |  |  |  | <0.001 |
| Median (IQR) | 69 (53, 78) | 70 (56, 81) | 70 (57, 81) | 70 (58, 81) |  |
| **Age group, year** |  |  |  |  | <0.001 |
| 65+ years | 3329/5773 (58%) | 4526/7578 (60%) | 5270/8505 (62%) | 6678/10473 (64%) |  |
| Adults (18 - 64 years) | 2444/5773 (42%) | 3052/7578 (40%) | 3235/8505 (38%) | 3795/10473 (36%) |  |
| **Location of patient in hospital on survey day** | | |  |  |  |
| Medicine | 2630/5773 (46%) | 3236/7578 (43%) | 3436/8505 (40%) | 4619/10465 (44%) | <0.001 |
| Surgery | 2117/5,773 (37%) | 2538/7578 (33%) | 2124/8505 (25%) | 2461/10465 (24%) | <0.001 |
| Adult ICU | 293/5773 (5.1%) | 490/7578 (6.5%) | 583/8505 (6.9%) | 705/10465 (6.7%) | <0.001 |
| Hematology/Oncology/Bone Marrow Transplant | 251/5773 (4.3%) | 313/7578 (4.1%) | 422/8505 (5.0%) | 589/10465 (5.6%) | <0.001 |
| Critical/coronary care (not ICU) | 168/5773 (2.9%) | 187/7578 (2.5%) | 327/8505 (3.8%) | 602/10465 (5.8%) | <0.001 |
| Trauma/burn | 98/5773 (1.7%) | 87/7,578 (1.1%) | 107/8505 (1.3%) | 154/10465 (1.5%) | 0.031 |
| Solid organ transplant | 83/5773 (1.4%) | 160/7,578 (2.1%) | 71/8505 (0.8%) | 130/10465 (1.2%) | <0.001 |
| **Additional precautions** | 390/5773 (6.8%) | 1,125/7,578 (15%) | 1,311/8505 (15%) | 2,272/10473 (22%) | <0.001 |
| Contact precautions | 244/5773 (3.9%) | 1029/7,578 (13.6%) | 1189/8505 (14.0%) | 2120/10473 (20.2) | <0.001 |
| Droplet/contact precautions | 6/5773 (0.1%) | 45/7578 (0.6%) | 228/8505 (2.7%) | 451/10473 (4.3%) | <0.001 |
| Airborne precautions | 17/5773 (0.3%) | 53/7578 (0.7%) | 39/8505 (0.5%) | 60/10473 (0.6%) | 0.009 |
| **Invasive device** | N/A | N/A | N/A | 2526/6644 (38%) | N/A |
| IQR = interquartile range; ICU = intensive care unit | | | | | |

**Supplementary Table 4.** Prevalence of healthcare-associated infections among adult inpatients and ICU patients surveyed in 2002, 2009, 2017 and 2024

|  | **Overall (2002-2024)**  **N=32,329** | | | **2002 N=5,773** | | | **2009 N=7,578** | | | **2017 N=8,505** | | | **2024 N=10,473** | | | ***P* value** |
| --- | --- | --- | --- | --- | --- | --- | --- | --- | --- | --- | --- | --- | --- | --- | --- | --- |
| **All adult inpatients** | **n** | **%** | **95% CI** | **n** | **%** | **95% CI** | **n** | **%** | **95% CI** | **n** | **%** | **95% CI** | **n** | **%** | **95% CI** |  |
| All HAIs surveyed | 3,099 | 9.6 | 9.3-9.9 | 601 | 10.4 | 9.6-11.2 | 939 | 12.4 | 11.7-13.2 | 715 | 8.4 | 7.8-9.0 | 844 | 8.1 | 7.6-8.6 | <0.001 |
| UTI | 1014 | 3.1 | 3.0-3.3 | 208 | 3.6 | 3.1-4.1 | 362 | 4.8 | 4.3-5.3 | 235 | 2.8 | 2.4-3.1 | 209 | 2.2 | 1.7-2.5 | <0.001 |
| Pneumonia | 796 | 2.5 | 2.3-2.6 | 176 | 3.0 | 2.6-3.5 | 220 | 2.9 | 2.5-3.3 | 168 | 2.0 | 1.7-2.3 | 232 | 2.2 | 2.0-2.5 | <0.001 |
| SSI | 665 | 2.1 | 1.9-2.2 | 147 | 2.5 | 2.2-3.0 | 182 | 2.4 | 2.1-2.8 | 166 | 2.0 | 1.7-2.3 | 170 | 1.6 | 1.4-1.9 | <0.001 |
| BSI | 464 | 1.4 | 1.3-1.6 | 93 | 1.6 | 1.3-2.0 | 127 | 1.7 | 1.4-2.0 | 85 | 1.0 | 0.8-1.2 | 159 | 1.5 | 1.3-1.8 | <0.001 |
| CDI | 301 | 0.9 | 0.8-1.0 | 59 | 1.0 | 0.8-1.3 | 97 | 1.3 | 1.0-1.6 | 71 | 0.8 | 0.7-1.1 | 74 | 0.7 | 0.6-0.9 | <0.001 |
| VRI | 88/18978 | 0.5 | 0.4-0.6 | n/a | | | n/a | | | 23 | 0.3 | 0.2-0.4 | 65 | 0.6 | 0.5-0.8 | <0.001 |
| Viral gastroenteritis | 11/18978 | 0.1 | 0.03-0.1 | n/a | | | n/a | | | 7 | 0.1 | 0.04-0.2 | 4 | <0.1 | 0.01-0.1 | 0.01 |

|  | **Overall (2017-2024)**  **N=18,978** | | | **2002** | | | **2009** | | | **2017 N=8,505^1^** | | | **2024 N=10,473^1^** | | | ***P* value** |
| --- | --- | --- | --- | --- | --- | --- | --- | --- | --- | --- | --- | --- | --- | --- | --- | --- |
| **All adult inpatients** | **n** | **%** | **95% CI** | **n** | **%** | **95% CI** | **n** | **%** | **95% CI** | **n** | **%** | **95% CI** | **n** | **%** | **95% CI** |  |
| CAUTI | 204 | 1.1 | 0.9-1.2 | n/a | | | n/a | | | 101 | 1.2 | 1.0-1.5 | 103 | 1.0 | 0.8-1.2 | 0.18 |
| VAP | 131 | 0.7 | 0.6-0.8 | n/a | | | n/a | | | 53 | 0.6 | 0.5-0.8 | 78 | 0.7 | 0.6-0.9 | 0.31 |
| CLABSI | 108 | 0.6 | 0.5-0.7 | n/a | | | n/a | | | 36 | 0.4 | 0.3-0.6 | 72 | 0.7 | 0.-0.9 | **0.02** |
| SSI with implant | 90 | 0.5 | 0.4-0.6 | n/a | | | n/a | | | 46 | 0.5 | 0.4-0.7 | 44 | 0.4 | 0.3-0.6 | 0.23 |
|  | **Overall (2002-2024)**  **N=2,071** | | | **2002 N=293^1^** | | | **2009 N=490^1^** | | | **2017 N=583^1^** | | | **2024 N=705^1^** | | | ***P* value** |
| **Adult ICU patients** | **n** | **%** | **95% CI** | **n** | **%** | **95% CI** | **n** | **%** | **95% CI** | **n** | **%** | **95% CI** | **n** | **%** | **95% CI** |  |
| All HAIs surveyed | 512 | 24.7 |  | 104 | 35.5 | 30.1-41.3 | 145 | 26.9 | 25.6-34.0 | 118 | 20.2 | 17.1-23.8 | 145 | 20.6 | 17.7-23.8 | <0.001 |
| Pneumonia | 303 | 14.6 | 13.2-16.2 | 60 | 20.5 | 16.1-25.6 | 85 | 17.3 | 14.2-21.1 | 71 | 12.2 | 9.7-15.2 | 87 | 12.3 | 10.1-15.1 | <0.001 |
| UTI | 81 | 3.9 | 3.4-4.9 | 19 | 6.5 | 4.1-10.1 | 32 | 6.5 | 4.6-9.2 | 9 | 1.5 | 0.8-3.0 | 21 | 3.0 | 1.9-4.6 | <0.001 |
| BSI | 108 | 5.2 | 4.3-6.3 | 31 | 10.6 | 7.4-14.8 | 24 | 4.9 | 3.2-7.3 | 12 | 2.1 | 1.1-3.7 | 41 | 5.8 | 4.3-7.9 | <0.001 |
| CDI | 27 | 1.3 | 0.9-1.9 | 5.0 | 1.7 | 0.6-4.2 | 4 | 0.8 | 0.3-2.2 | 12 | 2.1 | 1.1-3.7 | 6.0 | 0.9 | 0.4-1.9 | 0.18 |
| SSI | 73 | 3.5 | 2.8-4.4 | 20 | 6.8 | 4.3-10.5 | 17 | 3.5 | 2.1-5.6 | 20 | 3.4 | 2.2-5.3 | 16 | 2.3 | 1.4-3.7 | 0.005 |
| VRI | 8/1288 | 0.6 | 0.3-1.3 | n/a | | | n/a | | | 4 | 0.7 | 0.2-1.9 | 4 | 0.6 | 0.2-1.6 | 0.5 |
| Viral gastroenteritis | 1/1288 | 0.1 | 0.004-0.5 | n/a | | | n/a | | | 1 | 0.2 | 0.01-1.1 | 0 | 0 | 0 | 0.66 |

UTI = urinary tract infection; BSI = bloodstream infection; CDI = *Clostriodioides* difficile infection; SSI = surgical site infection; VRI = viral respiratory infection; CAUTI = catheter-associated urinary tract infection; VAP = Ventilator-associated pneumonia; CLABSI = central line associated bloodstream infection; SSI with implant = surgical site infection associated with a prosthetic implant; n/a = not available (data not collected)

**Supplementary Table 5**. Prevalence of invasive devices among adult inpatients by ward, 2024.

| **Characteristic** | **Adult ICU N=460^1^** | **95% CI^2^** | **Coronary Care  N=329^1^** | **95% CI^2^** | **Medicine N=2964^1^** | **95% CI^2^** | **Surgery N=1477^1^** | **95% CI^2^** | **p-value** |
| --- | --- | --- | --- | --- | --- | --- | --- | --- | --- |
| **Invasive device** | 412/460 (89.6%) | 86.3%, 92.1% | 71/329 (21.6%) | 17.3%, 26.5% | 845/2,964 (28.5%) | 26.9%, 30.2% | 588/1,477 (39.8%) | 37.3%, 42.4% | **<0.001^3^** |
| **Indwelling urinary catheter** | 278/460 (60.4%) | 55.8%, 64.9% | 42/329 (12.8%) | 9.45%, 17.0% | 432/2,964 (14.6%) | 13.3%, 15.9% | 226/1,477 (15.3%) | 13.5%, 17.3% | **<0.001^3^** |
| **Central venous catheter** | 306/460 (66.5%) | 62.0%, 70.8% | 29/329 (8.8%) | 6.08%, 12.5% | 312/2,964 (10.5%) | 9.46%, 11.7% | 214/1,477 (14.5%) | 12.8%, 16.4% | **<0.001^3^** |
| **Inserted tubes and drains** | 221/460 (48.0%) | 43.4%, 52.7% | 20/329 (6.1%) | 3.85%, 9.38% | 242/2,964 (8.2%) | 7.22%, 9.22% | 293/1,477 (19.8%) | 17.9%, 22.0% | **<0.001^3^** |
| **Endotracheal intubation** | 173/460 (37.6%) | 33.2%, 42.2% | 5/329 (1.5%) | 0.561%, 3.72% | 46/2,964 (1.6%) | 1.15%, 2.08% | 37/1,477 (2.5%) | 1.79%, 3.47% | **<0.001^3^** |
| ^1^n/N (%) | | | | | | | | | |
| ^2^CI = Confidence Interval | | | | | | | | | |
| ^3^Pearson's Chi-squared test | | | | | | | | | |

**Supplementary Table 6**. Number and proportion of microorganisms by survey year, 2002, 2009, 2017 and 2024.

| **Microorganism** | **2002**​  N=570 | **2009**​  N=792 | **2017**  N=592 | **2024**  N=747 | ***P* value** |
| --- | --- | --- | --- | --- | --- |
| *E. coli* | 84 (14.7%) | 171 (21.6%) | 117 (19.8%) | 126 (16.9%) | 0.006 |
| *Enterococcus spp.* | 101 (17.7%) | 115 (14.5%) | 77 (13.0%) | 112 (15.0%) | 0.15 |
| *Staphylococcus aureus* | 81 (14.2%) | 99 (12.5%) | 94 (15.9%) | 95 (12.7%) | 0.25 |
| *Klebsiella spp.* | 28 (4.9%) | 67 (8.5%) | 54 (9.1%) | 64 (8.6%) | 0.03 |
| *Pseudomonas spp.* | 45 (7.9%) | 63 (8.0%) | 42 (7.1%) | 55 (7.4%) | 0.93 |
| *Candida spp.* | 53 (9.3%) | 37 (4.7%) | 45 (7.6%) | 41 (5.5%) | 0.003 |
| *Enterobacter spp.* | 21 (3.7%) | 31 (3.9%) | 25 (4.2%) | 35 (4.7%) | 0.81 |
| *SARS-CoV-2* | n/a | n/a | n/a | 28 (3.7%) | n/a |
| *Other staphylococcus spp.* | 69 (12.1%) | 56 (7.1%) | 31 (5.2%) | 30 (4.0%) | <0.001 |
| *Proteus spp.* | 5 (0.9%) | 14 (1.8%) | 10 (1.7%) | 18 (2.4%) | 0.22 |
| *Streptococcus spp.* | 20 (3.5%) | 40 (5.1%) | 20 (3.4%) | 26 (3.5%) | 0.28 |
| Other | 63 (11.0%) | 99 (12.5%) | 77 (13.0%) | 117 (15.7%) | 0.09 |

n/a = not available (data not collected)
